# Academic Global Surgery: Creating Opportunities, Equity, and Diversity

**DOI:** 10.5334/aogh.3972

**Published:** 2023-02-14

**Authors:** Tanaz Vaghaiwalla, Sandesh Gyawali, Anusha Jayaram, Priyansh Nathani, Riya Sawhney, Kristin Long, Christopher Dodgion, Nakul Raykar, Juan Carlos Puyana, Anip Joshi, The Advocacy Committee of the Association for Academic Global Surgery

**Affiliations:** 1 Department of Surgery, University of Tennessee Graduate School of Medicine, Knoxville, TN, US; 2 Ascension Saint Francis Hospital, Evanston, IL, US; 3 Beth Israel Deaconess Medical Center, Boston, MA, US; 4 WHO Collaborating Centre for Research in Surgical Care Delivery in Low– and Middle–Income Countries, IN; 5 Program in Global Surgery and Social Change Department of Global Health and Social Medicine, Harvard Medical School, Boston, Massachusetts, US; 6 University of Pittsburgh, Professor of Surgery, Critical Care Medicine, and Clinical Translational Science, US; 7 Department of Surgery, Bir Hospital, National Academy of Medical Sciences, NP

**Keywords:** Academic Global Surgery, Global Surgery, Global Health, Health Equity, Capacity-building

## Abstract

A workforce trained in the development and delivery of equitable surgical care is critical in reducing the global burden of surgical disease. Academic global surgery aims to address the present inequities through collaborative partnerships that foster research, education, advocacy and training to support and increase the surgical capacity in settings with limited resources. Barriers include a deficiency of resources, personnel, equipment, and funding, a lack of communication, and geographical challenges. Multi–level partnerships remain fundamental; these types of partnerships include a wide range of trainees, professionals, institutions, and nations, yet care must be taken to avoid falling into the trap of surgical “voluntourism” and undermining the expertise and practice of long–standing frontline providers. Academic global surgery has the benefit of developing a community of surgeons who possess the tools needed to collaborate on individual, institutional, and international levels to address inequities in surgery that are spread variously across the globe. However, challenges for surgeons pursuing a career in global surgery include balancing clinical responsibilities while integrating global surgery as a career during training. This is due in part to the lack of mentorship, research time, grant funding, support to attend conferences, and a limitation of resources, all of which are significantly more pronounced for surgeons from low–resource countries.

## Introduction to Academic Global Surgery

Global surgery is a rapidly growing field aimed at delivering equitable surgical care worldwide. Academic global surgery seeks to address current inequities through partnerships promoting research, education, advocacy, and training, often across academic departments and institutions. The advancement and delivery of surgical and anesthetic care in low– and lower middle–income countries (LICs and LMICs) has been a low priority on the global health stage until recently, with limited representation in global health literature and policy forums. The lack of human resources, accessory equipment, geographical accessibility, and means for communication all contribute to disparities in care [1]. Thoughtfully crafted academic collaborations—cognizant of historical power dynamics and abuses within partnerships between LICs/LMICs and high–income countries (HICs)—are integral in advancing the vision of universal access to high–quality surgical care. Sustainable programming includes capacity–building and training endeavors to support and increase the global surgery workforce, as well as the professional development of clinicians and researchers in low–resource settings [1,2].

## Academic Global Surgery Advances the Health Equity Agenda

Barriers to providing equitable surgical care include the deficiency of resources, personnel, equipment, funding, a lack of communication, and geographical challenges. Academic global surgery fosters a collaborative community that identifies disparities through research, education, and programming between institutions. It aims to empower frontline physicians in all contexts through academic collaboration across innovation, implementation, and dissemination, to decrease healthcare inequities. As an example, The Global Financing Facility for Women, Children and Adolescents (GFF) is a global partnership that supports 36 LIC and LMICs. GFF aims to aid the development and implementation of prioritized national health plans in order to scale–up access to affordable, quality care for women, children, and adolescents. GFF utilizes multi–level partnerships with governments, civil society organizations, UN agencies, Gavi, the Vaccine Alliance, the Global Fund, and the private sector. Together they have used finance value chains including resource mobilization, pooling, channeling, and resource allocation and implementation, and combined these steps to direct a large amount of funding toward addressing HIV/AIDS, malaria, tuberculosis, and vaccine–preventable diseases in LMICs [3,4]. Another example is the Himalayan Cataract Project (HCP), a non–governmental organization founded by two ophthalmologists in 1994. HCP provides cost–effective cataract surgery in Nepal by making intraocular lenses for $4, compared to higher–priced lenses produced in high–resource countries. To date, HCP has provided 13,000,000 eye health screenings and 1,015,000 cataract surgeries to patients in 18 LIC and LMICs since the program’s initiation in 1995. HCP also builds sustainable eye healthcare systems through the education and training of local providers, ensuring the longevity of the initiative. From 1994 to 2016, 19,381 ophthalmic personnel from 43 countries received training in the primary training facility of HCP at the Tilganga Institute of Ophthalmology in Kathmandu, Nepal [5]. Investing in and learning from models like GFF and HCP should result in the reduction of healthcare costs, improving its affordability for uninsured and disadvantaged populations. These efforts within academic global surgery support and improve both the quality and quantity of surgical care, ultimately contributing to the improvement of patient care worldwide. Academic global surgery aims to address challenges such as gender gaps and regional underrepresentation through advocacy and opportunities that create an equitable surgical workforce and allow diverse authorship, editorial boards, and leadership.

## Academic Global Surgery and Capacity-Building

Many global surgery publications describe surgeons and trainees from HICs traveling for elective rotations during their training, or even undertaking shorter duration trips to LMICs. With increased awareness of global health inequity, there has been a greater spike in the promotion of sustainable and equitable bilateral exchanges between partners [6]. The aim of academic global surgery is to support and increase the surgical capacity in resource–limited settings. However, care must be taken to avoid falling into the trap of ‘voluntourism’, which has the potential to supersede the efforts and expertise of Long-term frontline providers or create distrust between providers and the public in that region [7]. HIC physicians who participate in projects such as educational rotations or medical mission trips should be aware of their limitations and acknowledge that their involvement may, in fact, be disruptive. Exchanges should be bilateral and include clear goals outlined by both partners, whether they be educational, clinical, or involve other objectives. Collaborations help ensure that efforts are symbiotic and beneficial for both groups, including opportunities for clinical training, research, education, and advocacy. One model of longitudinal and bilateral partnerships is the Haitian Orthopedic Residency Exchange Program, where orthopedic teams from South Carolina, funded by the South Carolina Orthopedic Association (SCOA), developed a training program to address the musculoskeletal needs of the Haitian people and improve the overall standard of orthopedic training in Haiti. Orthopedic attendings and residents travel to Haiti from the United States bimonthly and work alongside Haitian attendings and residents, while Haitian surgical residents travel to the United States as part of their resident education. Since 2014, the program has facilitated the treatment of over 2000 orthopedic patients in the clinical setting, supervised over 650 inpatients, and performed 554 surgeries [8].

In addition to clinical exchanges and education programs, technology can be leveraged to enhance local human resources. An example of using innovative technology is Virtual Reality in Medicine and Surgery (VRIMS), which aims to bring affordable virtual reality technology to medical, surgical, and dental trainees for clinical demonstrations of techniques and simulation training [9]. As surgical pathologies may differ between geographical locations, there may be mutual benefits from bilateral exchanges, particularly with respect to surgical education and skill sets.

## Mutual Benefits of Bilateral Exchange

For surgeons training in HICs, challenges remain when it comes to balancing clinical time and demands while integrating academic global surgery as a career during training, due to lack of mentors, research time, grant funding, support with conference attendance, and limited resources [10,11]. These surgeons from HICs have the unique opportunity to gain surgical experience, where the focus is driven towards clinical evaluation rather than an overreliance on imaging techniques and towards procedural techniques, rather than minimally invasive alternatives. Surgeons may gain a new awareness of cultural differences in the delivery of patient care or, could similarly brainstorm cost-effective strategies unique to the region Where they work.

With these potential, more conventionally recognized benefits for HIC surgeons visiting LIC or LMICs, the onus of ensuring a mutually beneficial partnership also lies with them. It is imperative that they defer to their LMIC partners to set the agenda and the goals they wish to accomplish during the exchange. For example, surgeons or trainees from LMICs may not be interested in learning about certain technological advances that are not applicable or available in their region. Therefore, it is crucial for surgeons from HICs to create safe and equitable spaces to establish mutual expectations with their LMIC colleagues. Furthermore, it would be remiss not to emphasize that a true ‘bilateral’ exchange would also involve a converse learning opportunity for LMIC surgeons in partner HIC locations. An example of a bilateral and sustainable partnership is the American College of Surgeons (ACS) and the College of Surgeons of East, Central, and Southern Africa (COSECSA) Surgical Training Collaborative, which has created programs to increase the number of trained surgeons in the region, where the ratio of surgeons to the population is 0.53 surgeons to 100,000 people [7,12]. The Surgical Training Collaborative at Hawassa University in Ethiopia aims to address this critical shortage through capacity building and by increasing the local surgical workforce. Training is delivered by visiting faculty from the US, who teach and mentor surgeons year–round at Hawassa with the goal of increasing capacity to address the region’s increasingly complex surgical care needs [7]. Bilateral partnerships increase awareness of each region’s unique requirements through collaboration with frontline researchers, institutions, communities, and governments, while developing an international community of surgeons with the tools to address inequities in global surgery [13,14].

## Academic Global Surgery Creates Opportunities for a Diverse Group of Surgeons

Historical colonial and hierarchical influences continue to permeate the global health field. Due to the lack of equitable promotion and recognition, women often fall behind men when it comes to attaining academic positions, editorial board leadership, and research grant approval [15,16]. While women make up nearly 50% of medical school graduates in the US, they make up only 21% of general surgeons and 10% of full professors [17,18]. While research suggests that progress has been made in ensuring that authorship reaches parity, a lack of gender equity and regional inclusion within authorship and editorial leadership in global surgery remains [15,16,19,20,21,22]. These disparities are even more pronounced among those from low–resource countries. A systematic analysis found that authorship within the global surgical literature was dominated by male authors and authors affiliated with HICs. A gender gap was identified among the LMIC authors, with a lower proportion of female compared to male authors at nearly every seniority grade of authorship, and there was a lack of LMIC authors in the first and senior author positions [16,19]. Another study of 12 major global health journals and 551 editors found that 35% of editors were women. When examining distribution regionally, only 33% of the editors were based in LMICs overall. Women based in LMICs and women in leadership roles from LMICs represented only 11% and 4% of all editors, respectively [22]. Based on the current rate of progress, it is estimated that women will not achieve equity in academic surgical positions, particularly full professors, for over a century [17]. Academic global surgery aims to address these gender gaps and regional underrepresentation through advocacy and through opportunities that create an equitable surgical workforce and allow for greater diversity in authorship, editorial boards, and leadership.

## Continued Barriers/Need for Global Surgery Competencies

Barriers to equity in the global surgery workforce may include a lack of an educational framework of competencies among academic programs, the inaccessibility of educational content, or a shortage of research opportunities and bilateral partnerships [11,12,13]. One study evaluating global surgery research in rural Rwanda identified several potential barriers, such as a limited number of experienced investigators trained in global surgery research, time constrictions faced by researchers, and journal regulations. The study highlighted the need for collaboration in all areas of global surgery research to allow sharing of opportunities and partnerships between LMICs and HICs and thereby enhance the impact of the research [13]. Another study evaluated whether the published literature for a global surgery curriculum aligned with current competency frameworks in global health and surgical education from the Consortium of Universities for Global Health (CUGH) and Accreditation Council for Graduate Medical Education (ACGME). The results of this study showed no consensus on global surgery competencies among institutional curricula, and the majority of the literature (17 of 18 eligible studies) were from and tailored to HICs. With no universally accepted framework among global health institutions, the development of core competencies could ensure a workforce adequately trained in global surgery. Accessibility is equally vital to ensure that education and resources are available to all partners in global surgery [23].

Finally, multi–level collaborative partnerships remain fundamental and include a wide range of individuals, including trainees, professionals, institutions, and nations. For example, global health conferences are essential forums for academic exchange, policy making, and professional development. However, historically, global health conferences are hosted and planned by leadership within HICs, and attendees from LMICs have been grossly Under-represented. A systematic review evaluating LMIC representation at a total of 112 global health conferences found only 4% of conferences were hosted in LICs. Conference equity also has systemic challenges, of which the authors cited travel expenses, visa constraints, and lower rates of acceptance for research presentations and speaker invitations. Proposed solutions included meeting relocation to visa–friendly countries, travel grants, and mentorship programs to facilitate the inclusion of LMIC researchers at global health meetings [24]. An example of a multi–level and multi–disciplinary collaborative effort is the WHO Collaborating Centre for research in surgical care delivery in LMICs, based in India, which aims to build an inclusive consortium and network for LMIC researchers from across the globe. Its goal is to create a network of people with different skill sets to work as teams providing cross–learnings and peer–to–peer education to build research capacity and scholarship that cuts through hierarchy, language, socio–cultural and geographical barriers [25].

Academic global surgery fosters research collaboration by providing avenues for interested LMIC institutions to engage in bilateral endeavors with HICs, accessible health registries, support through grants, and mentorship. Incorporating global health and public health courses into the process may bolster interest in global surgery as a career. Furthermore, academic global surgery seeks to increase opportunities supporting surgical training programs in LMICs. Teaching didactic sessions, supervising residents, and mentoring can contribute to the academic portfolio of HIC surgeons during their promotions. In the same way, many LMIC surgeons can be mentors for future surgeons in their home countries. The field will also pave the way forward for interdisciplinary and interprofessional alliances with health and non–health related professions like health economics, health policies, biomedical engineering and health information technology, and benefit both health and non–health related professions. In addition, the representation of local surgeons from LMICs in publications and conferences has been lacking in global surgical efforts. Some studies and editorials have emphasized directions to ensure inclusivity in publications through suggestions on authorship guidelines, both to ensure more equitable representation and acknowledge the contributions of local stakeholders in global health projects [26].

## Conclusions

A workforce trained in the development and delivery of equitable surgery remains integral to reducing the global burden of surgical disease. Academic global surgery has the benefit of developing a community of surgeons that possess the tools needed to collaborate on individual, institutional, and international levels to address the inequities in surgical practices that are spread variously across the globe. Long–term goals of academic global surgery include sustainable capacity–building and efforts to strengthen healthcare systems through the bilateral, mutually beneficial exchange of knowledge, skills, and resources.
